# Antiaging function of Chinese pond turtle (*Chinemys reevesii*) peptide through activation of the Nrf2/Keap1 signaling pathway and its structure-activity relationship

**DOI:** 10.3389/fnut.2022.961922

**Published:** 2022-07-22

**Authors:** Qianqian Wang, Zherui Yang, Jiachen Zhuang, Junhui Zhang, Fei Shen, Peng Yu, Hao Zhong, Fengqin Feng

**Affiliations:** ^1^College of Biosystems Engineering and Food Science, Zhejiang University, Hangzhou, China; ^2^Yuyao Lengjiang Turtle Industry, Ningbo, China; ^3^College of Food Science and Technology, Zhejiang University of Technology, Hangzhou, China

**Keywords:** Chinese pond turtle peptide, antioxidant, antiaging, Nrf2/Keap1 pathway, molecular docking

## Abstract

Chinese pond turtle is a traditional nourishing food with high nutritional value and bioactivity and has been considered a dietary remedy for prolonging the lifespan since ancient times. However, only limited information about their effects on longevity is available. This study was performed to assess the antioxidant activities and antiaging potential of Chinese pond turtle peptide (CPTP) using *Drosophila melanogaster* model and uncover the possible mechanisms underlying the beneficial effects. CPTP exhibited excellent antioxidant capability *in vitro* with *IC*_50_ values of 3.31, 1.93, and 9.52 mg/ml for 1,1-diphenyl-2-pycryl-hydrazyl (DPPH), 2,2-azinobis (3-ethylbenzothiazo-line-6-sulfonic acid) diammonium salt (ABTS), and hydroxyl radical scavenging, respectively. *In vivo*, 0.8% of CPTP significantly extended the mean and median lifespan of female flies by 7.66 and 7.85%, followed by enhanced resistance to oxidative and heat stress. Besides, CPTP remarkably increased the antioxidant enzyme activities and diminished the peroxide product accumulation. Furthermore, CPTP upregulated the relative mRNA expression of antioxidant-related genes, including nuclear factor-erythroid-2-like 2 (*Nrf2*) and its downstream target genes, while downregulated the expression of Kelch-like ECH-associated protein 1 (*Keap1*). Taken together, CPTP displayed promising potential in both antioxidant and antiaging effects on flies by targeting the Nrf2/Keap1 pathway. Further peptide sequence determination revealed that 89.23% of peptides from the identified sequences in CPTP could exert potential inhibitory effects on Keap1. Among these peptides, ten representative peptide sequences could actively interact with the binding sites of Keap1-Nrf2 interaction through hydrogen bonds, van der Walls, hydrophobic interactions, and electrostatic interactions. Conclusively, CPTP could be utilized as health-promoting bioactive peptide with antioxidant and antiaging capacities.

## Introduction

Aging, an inexorable biological process concomitant with the human body growing old, is characterized by incorrigible damage in the cell structure and failure in the functions of cells and tissues, which will ultimately lead to organismal death throughout the body (1). Researchers have been continuously studied the underlying mechanisms of aging for decades, and oxidative stress is well defined as one of the most possible causes. Oxidative stress occurs from the imbalance between the oxidative and antioxidative systems of cells and tissues, which involves the excessive generation of radicals such as the reactive oxygen species and reactive nitrogen species (2). These radicals can subsequently be deleterious to the stability of biomolecules such as proteins, lipids, and nucleic acids, thereby disrupting the transduction of redox signals and consequently causing molecular damage. To safeguard the tissues from oxidative damage, the body cultivated a potent defense system containing multiple antioxidant enzymes that can effectively exterminate the free radicals. Nuclear factor-erythroid-2-like 2 (Nrf2), a pivotal regulator of the activities of multiple antioxidant enzymes, monitors the status of cellular oxidative stress and further modulates the cellular redox balance (2). The deletion of the Nrf2 genes can disrupt the transcriptomic profiles, which are tightly linked to the occurrence of age-related diseases (3). Furthermore, aging is also well-connected with the loss of Nrf2 activity as evidenced by the impaired Nrf2 signaling in the retinal pigment epithelium of aging mice (4) and declines in stress resistance abilities and life span (5). The negative feedback loop is mainly regulated by the Kelch-like ECH-related protein 1 (Keap1), the complex of which contains the binding site for Cullin-dependent E3 ubiquitin ligase to degrade Nrf2. Nrf2 is commonly present in the cytoplasm binding to the Keap1 forming the Nrf2-Keap1 complex. While cellular oxidative stress occurs, Nrf2 is separated from Keap1 and then binds to the transcription binding site of multiple antioxidant enzymes to start the transcriptions (6). Nrf2 inducers, e.g., tert-butylhydroquinone and itaconate had the ability to react with cysteine thiol groups in Keap1 and further trigger the antioxidant responses (7, 8). Therefore, seeking candidate agents to inhibit oxidative stress by activating Nrf2 signaling could be crucial to delay aging and treat aging-related diseases.

Currently, studies are focused on discovering the antiaging benefits of bioactive antioxidants in different organisms. Notwithstanding, the antiaging effect conferred by antioxidants still remains elusive in different organisms and even adverse effects of specific antioxidants in human (9). As such, it is in great need for the discovery of safe antioxidants with the property of effective prevention and treatment of diseases induced by oxidative stress. Among them, bioactive peptides demonstrated excellent potential as health-promoting supplements concerning their versatile functions, extensive raw material sources, high specificity, and less side effects. To date, the major food sources of peptides with antioxidant activity are cereals (10), vegetables (11), eggs (12), nuts (13), milk (14), aquatic species (15), and so forth. Antioxidant peptides not only exhibited potent free radical scavenging activity and inhibiting lipid oxidation *in vivo* but also ameliorated reactive oxygen species-mediated lesions in cell and animal models (16). Intriguingly, several peptides have been reported to competitively binding to Keap1 in the substitution of Nrf2 by molecular docking and consequently inhibiting the formation of the Keap1-Nrf2 complex (17). Molecular docking is an effective structure-based method to screen out the potential bioactive compounds for new drug discovery and design by predicting the interaction between the receptor and ligand (18). For example, Wang et al. revealed that the antioxidant peptide from soft-shelled turtle hydrolysate could bond with the site of the Kelch domain (19). Additionally, Tyr-Ser-Asn-Gln-Asn-Gly-Arg-Phe from cottonseed proteins had a capacity to inhibit oxidative stress *in vivo* by forming intense molecular interactions with the Keap1 bind site (20).

Chinese pond turtle (*Chinemys reevesii*) is a commercially valuable edible aquatic species with rich protein sources. According to the traditional remedy claims by oriental medicine in China, the Chinese pond turtle may have a positive impact on the promotion of longevity. The previous study reported that the Chinese pond turtle hydrolysates contain high content of antioxidant components and antidiabetic activities (21). In addition, peptide extracted from soft-shelled turtle not only possessed superior antioxidant activity *in vitro* but also could regulate the Nrf2-mediated signaling pathway in mice (19, 22). These studies mentioned above lay the solid foundation of the potential utilization of Chinese pond turtle peptide (CPTP) as the antioxidant to deal with aging issues. Henceforth, the study of whether CPTP could diminish oxidative damage associated with aging is of great interest. However, there are few reports dealing with the possible antiaging effect of CPTP and the underlying mechanism.

*Drosophila melanogaster* has been developed as a potent model system to study dietary interventions, metabolic disorders, and aging due to its short lifespan, low cost, and ease of genetic manipulation, as well as the evolutionary conservation of central signaling pathways regulating metabolism and energy homeostasis (23–25). As such, in this study, the antioxidant capacity of CPTP was first measured *in vitro*, and then *Drosophila* was employed as a model organism to further explore the antiaging effect *in vivo* and to elucidate the potential mechanism. Furthermore, the peptide sequence of CPTP was determined and subsequently predicted the potential antioxidant peptides by molecular docking.

## Materials and methods

### Materials

Pond turtle protein-derived peptide (CPTP) were kindly provided by Hangzhou Kangyuan Food Science and Technology Co., Ltd. (Hangzhou, China). The molecular weight and amino acid composition of CPTP are shown in Supplementary Tables 1, 2. 1,1-Diphenyl-2-pycryl-hydrazyl (DPPH) and 2,2-azinobis (3-ethylbenzothiazo-line-6-sulfonic acid) diammonium salt (ABTS) were obtained from Sigma-Aldrich Company (St. Louis, MO, United States). Cornmeal, yeast, sucrose, and agar were purchased from the local market (Food grade, Hangzhou, China). All other chemicals used were procured from Sinopharm (Analytical grade, Shanghai, China).

### Antioxidant activity of Chinese pond turtle peptide *in vitro*

The DPPH radical scavenging activity was tested as previously reported with some modifications (26) and was calculated by the following formula:


$$\mathrm{DPPH}\;\mathrm{radical}\mathrm{scavenging}\mathrm{activity}\left(\%\right)=\frac{1-\left({\text{A}}_{\text{sample}}-{\text{A}}_{\text{reference}}\right)}{{\text{A}}_{\text{blank}}}$$

A volume of 80 μl of DPPH solution (0.1 mmol/L) was mixed with 100 μl CPTP solutions (1, 2.5, 5, 7.5, and 10 mg/ml).

The ABTS radical scavenging activity was determined as previously reported (27) and was calculated by the following formula:


$$\mathrm{ABTS}\;\mathrm{radical}\mathrm{scavenging}\mathrm{activity}\left(\%\right)=\frac{{\text{A}}_{\text{blank}}-{\text{A}}_{\text{sample}}}{{\text{A}}_{\text{blank}}}$$

A volume of 100 μl of CPTP solutions (0.5, 1, 2, 3, and 4 mg/ml) was blended with 100 μl of ABTS working solution (7 mmol/L).

The hydroxyl radical scavenging assay was performed as previously reported (28) and was calculated by the following formula:


$$\mathrm{Hydroxyl}\;\mathrm{radical}\mathrm{scavenging}\mathrm{activity}\left(\%\right)=\frac{1-\left({\text{A}}_{\text{sample}}-{\text{A}}_{\text{reference}}\right)}{{\text{A}}_{\text{blank}}}$$

A volume of 200 μl of FeSO_4_ (9 mmol/L), 200 μl of salicylic acid ethanol solution (9 mmol/L), 200 μl of CPTP solutions (5, 7.5, 10, 12.5, and 15 mg/ml), and 200 μl of H_2_O_2_ (0.03%, v/v) were mixed.

The reducing power assay was conducted as previously reported but with some modifications (29). The mixture of 1 ml of samples (5, 10, 15, 20, and 25 mg/ml), 500 μl of PBS (0.2 mol/L), 500 μl of K_3_Fe(CN)_6_ (1%, v/v), and 500 μl of CCl_3_COOH (10%, v/v) was blended and centrifuged. Then, 500 μl of supernatant was mixed with 500 μl of water and 100 μl of FeCl_3_ (0.1%, v/v). Subsequently, the absorbance was measured at 700 nm.

### Antiaging activity of Chinese pond turtle peptide *in vivo*

#### Fly husbandry and treatment

The wild-type Canton-S strain of *D. melanogaster* was acquired as a gift from the Institute of Food Bioscience and Technology of Zhejiang University and maintained at 25°C on a 12/12 h light-dark cycle at 65% humidity. The flies aged 3 days filled with the basal medium were named the young control group (NC). Female flies were cultured using basal medium or CPTP-supplemented medium for all experiments in this study. The basal medium was prepared according to the standard cornmeal medium containing cornmeal (10.5%, w/v), yeast (4%, w/v), sucrose (7.5%, w/v), agar (0.75%, w/v), and propionic acid (1%, v/v) (control group, CT). The CPTP-supplemented medium was added the peptide power into a cooled (65°C) liquid basal medium with the final concentration of 0.8% (w/w) (CPTP). To eliminate the possible parental effects, flies cultured for two generations were used for further experiments. Female flies were collected within 48 h from eclosion, sorted under CO_2_ anesthesia, and maintained at a density of 30–35 flies per bottle. The flies in the CT and CPTP groups were transferred to fresh food vials that contained various types of media every 3–4 days except for the NC group.

#### Lifespan analysis

Flies were maintained in the vial containing 3 ml of treatment medium (CT or CPTP) at a density of 30–35 flies, with ten replicate vials per treatment. The number of deaths was documented every 2 days until no survivors remained. The mean, median, and maximum life span were calculated by the following formula (30):


$$\mathrm{Mean}\mathrm{life}\text{span}\frac{\mathrm{Sum}\mathrm{of}\mathrm{survival}\mathrm{days}\mathrm{of}\mathrm{flies}}{\mathrm{Number}\mathrm{of}\mathrm{flies}}$$

MedianlifespanThedayofthesurvivaloffliesreached 50%

MaximumlifespanSumofsurvivaldaysofthelast 10% fliesNumberofthelast 10% flies

#### Feeding assay

The food intake was measured as previously reported (31). In brief, flies were transferred to 1% agar for 24 h of starvation at 30 days of age and subsequently cultured on the corresponding medium containing 0.5% (w/v) F&D blue No. 1 (Shanghai Macklin Biochemical Co., Ltd., Shanghai, China) for 4 h in the darkness. Then, flies were frozen in liquid nitrogen immediately, homogenized in 1 ml of PBS solution, and then get centrifuged. Later, 0.9 ml of supernatant was mixed with 0.6 ml of PBS and measured at 625 nm.

#### Stress assay

Flies for stress assays were reared and maintained for life span experiments at 30 days of age. For oxidative stress (30), flies in the two groups were transferred to a fresh vial containing a filter paper infiltrated by H_2_O_2_ solution (30%) with glucose (5%). The number of deaths was documented every 5 h, and H_2_O_2_ was supplemented once a day. For starvation analysis (32), flies were transferred to a fresh food vial with 1% agar. Similarly, deaths were documented every 5 h, with live flies transferred into fresh vials every 2–3 days. For heat stress (33), flies were transferred to an empty vial and incubated at 37°C, and deaths were documented every 0.5 h.

#### Determination of biochemical index

After 30 days of feeding, flies in two groups were frozen with liquid nitrogen and used for the activity of superoxide dismutase (SOD) and glutathione peroxidase (GSH-Px) and the content of malondialdehyde (MDA), protein carbonyl (PCO), and protein determination by the commercial kits according to the corresponding instructions (Nanjing Jiancheng Bioengineering Institute, Nanjing, China).

#### Real-time quantitative PCR

At 30 days of age, the total RNA was extracted from the whole flies (40 mg) using TRIzol reagent (Invitrogen, CA, United States), and then cDNA was synthesized with the HiScript III-RT SuperMix Kit (Vazyme Corp., Nanjing, China). After this, real-time quantitative PCR was conducted using SYBR Green PCR Master Mix on Light Cycle 480 (Roche Diagnostics, Penzberg, Germany). Equalized amplicons of ribosomal protein 49 (RP49) were utilized as a reference to normalize data through the method of 2^–ΔΔ*Ct*^. The primer sequences are available in Table 1.

**TABLE 1:** Sequences of primers used in this study.

Gene name	Forward primer (5′-3′)	Reverse primer (5′-3′)
*RP49*	GACAGTATCTGATGCCCAACA	CTTCTTGGAGGAGACGCCGT
*CncC*	GTCGCCACTAAAACCGCATC	TTGTTCTTTCCACGCCGACG
*Keap1*	GCGCTCGTCAGCCCATTTT	GGATGCGCATAATTCCTCTTCTT
*HO-1*	ATGACGAGGAGCAGCAGAAG	ACAAAGATTAGTGCGAGGGC
*Gclc*	GAGCCATTAGTGCCGTTAGT	GTCTTTCGTCTTCGTCTTGG

### Peptide sequence identification

The CPTP was desalted using a C18 stage tip before lyophilization according to the method of Hu et al. (16) and analyzed using the EASY-nLC-orbitrap MS/MS system. The desalted CPTP was dissolved in solvent A (0.1% formic acid in water) and subsequently loaded with C18 precolumn (100 × 2 mm, 3 μm, Acclaim PepMap). Then, the sample was separated by C18 column (75 × 25 mm, 3 μm, Acclaim PepMap) at a flow rate of 300 nl/min for 60 min with 5% mobile solvent B (0.1% formic acid in 100% acetonitrile) to 30% (Easy-nLC 1200 system, Thermo Scientific, Waltham, MA, United States). Later, the sample was subjected to a nano-ESI source followed by tandem mass spectrometry analysis in the Q-Exactive system. Finally, the peptide sequences were determined by *de novo* sequencing, and the sequences with the average local confidence (ALC) ≥ 90% were considered reliable.

### Molecular docking

In this study, the interaction between the Kelch domain of Keap1 (as the receptor, PDB ID: 2FLU) and peptides identified in the Section “Peptide sequence identification” (as the ligands) was performed using Discovery Studio software (Accelrys, San Diego, CA, United States) (20). The structure of Keap1 was optimized by cleaning, preparation, dewatering, and hydrogenation operation. The docking program was conducted with special binding sites (coordinates: *x* = 15.099270, *y* = 14.964499, *z* = 6.205664) and a set receptor radius (25.00 Å). The complex of Keap1-peptide with the highest (-) CDOCKER energy was regarded as the most stable conformation.

### Statistical analyses

All plotted values represent means ± SEM. GraphPad Prism 6.0 was used to perform the statistical analysis. *P*-value < 0.05 was considered statistically significant. Two-tailed unpaired *t*-test was used to analyze the comparisons between two independent groups, and one-way ANOVA with Tukey’s test was used for comparisons among three groups.

## Results and discussion

### Chinese pond turtle peptide exhibited antioxidant activity *in vitro*

**FIGURE 1:**
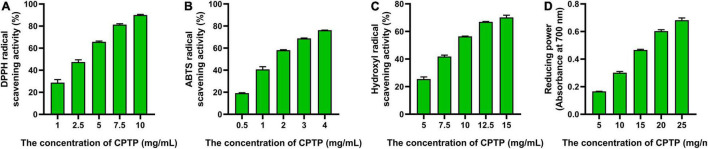
Antioxidant activities of Chinese pond turtle peptide (CPTP) *in vitro*. Radical scavenging activity of **(A)** 1,1-diphenyl-2-pycryl-hydrazyl (DPPH); **(B)** 2,2-azinobis (3-ethylbenzothiazo-line-6-sulfonic acid) diammonium salt (ABTS); **(C)** Hydroxyl; **(D)** Reducing power. The data are presented as mean ± SEM.

As shown in Figure 1, the half-maximal inhibitory concentrations (*IC*_50_) of CPTP to DPPH, ABTS, and hydroxyl were 3.31, 1.93, and 9.52 mg/ml, respectively, while the reducing power was 17.08 mg/ml at 700 nm when the absorbance value was 0.5. Cui et al. reported that the *IC*_50_ of DPPH radical scavenging activities from the four hydrolysates of milk protein concentrate with different proteases was 4.08, 10.24, 8.03, and 3.71 mg/ml, respectively (14). Similarly, it revealed that the ABTS radical scavenging activity of cottonseed protein-derived antioxidant peptides was 2.05 (20). Overall, CPTP exhibited an extraordinary ability to exterminate capture radicals and enhanced the reducing power, which suggested that CPTP had strong antioxidant activity *in vitro*.

### Chinese pond turtle peptide extended lifespan without limiting dietary intake in flies

**FIGURE 2:**
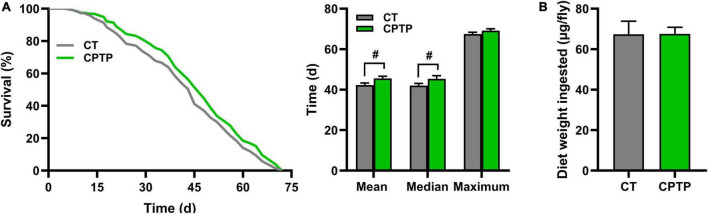
Chinese pond turtle peptide (CPTP) extended life span without changing food intake in female flies. **(A)** Life span curves, *n* = 300–350 flies per group; **(B)** Food intake, *n* = 300–350 flies per group. The data are presented as mean ± SEM. Statistical test: two-tailed unpaired *t*-test [^#^*P* < 0.05 vs. control group (CT) group].

Compared with the CT group, the mean lifespan, median lifespan, and maximum lifespan of the CPTP group were prolonged by 7.66% (from 42.32 to 45.56 days, *P* < 0.05), 7.85% (from 42.05 to 45.35 days, *P* < 0.05), and 2.54% (from 67.47 to 69.18 days, *P* = 0.1570) in flies, respectively (Figure 2A). That is, the mean lifespan and median lifespan were significantly increased, whereas the maximum lifespan was not affected by CPTP. Similarly, Moretti et al. reported that dietary nitrite extended the median lifespan but not the maximum lifespan (34) in female flies. Lifespan extension of flies has been hypothesized to be the result of either a slower aging process or a lower risk of death in likelihood caused by aging-related damage (35). Previous studies demonstrated that dietary restriction could extend lifespan both in vertebrates and invertebrates (36–38). Therefore, the food intake was tested to elucidate whether the lifespan-promoting of CPTP was indirectly caused by the self-imposed dietary restriction for the change of medium’s taste. Compared with the CT group, flies in the CPTP group only exhibited a mild increase in the average food consumption (Figure 2B). Thus, the extended longevity of CPTP appears to be independent of dietary restriction. Similarly, bioactivity peptides such as cultured crocodile meat hydrolysates (39) and crimson snapper peptides (40) were also shown to increase the survival curve without limiting food intake. Taken together, our results suggested that CPTP supplementation could extend lifespan without limiting food intake in flies.

### Chinese pond turtle peptide improved stress resistance in flies

**FIGURE 3:**
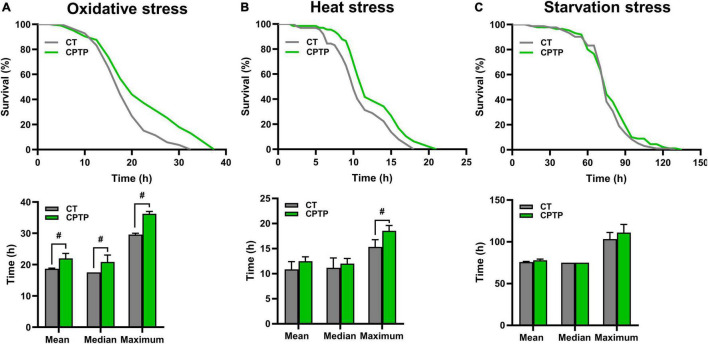
Chinese pond turtle peptide (CPTP) improved stress resistance in female flies. **(A)** Oxidative stress; **(B)** Heat stress; and **(C)** Starvation stress. *n* = 180–210 flies per group. The data are presented as mean ± SEM. Statistical test: two-tailed unpaired *t*-test (^#^*P* < 0.05 vs. CT group).

Stress tolerance and longevity are mechanistically and phenotypically closely linked (41). Generally, a longer life span in animal models has a better ability to resist environmental stress. To investigate whether CPTP could improve the resistance to stress, flies were pretreated with CPTP for 30 days, followed by exposure to oxidative stress (30% H_2_O_2_), heat stress (37°C), and starvation stress (1% agar), respectively. For the resistance to oxidative stress, flies were given H_2_O_2_ (a strong and unstable oxidant), which has been frequently utilized for the temptation of oxidative stress in numerous model organisms such as yeast (42), nematode (43), and *Drosophila* (44). Comparison with the CT group, CPTP treatment increased the survival rate of flies under oxidative stress, and the mean, median, and maximum lifespans were extended by 17.77% (*P* < 0.05), 19.05% (*P* < 0.05), and 22.54% (*P* < 0.05), respectively (Figure 3A). The results were in line with the antioxidant activity of CPTP *in vitro*. Furthermore, compared with the CT group, the tolerance to heat stress of flies in the CPTP group was almost the same as their effects against oxidative stress, where the extension of mean, median, and maximum lifespans by 15.14, 7.46, and 21.01% (*P* < 0.05), respectively (Figure 3B). However, it is unexpected that CPTP supplementation failed to enhance the resistance of flies to starvation stress (Figure 3C). This phenomenon was also discovered in the research by Su et al., which was probably associated with the lower triacylglyceride accumulation in flies (45). Therefore, we subsequently measured the fat storage and found that CPTP did not affect the triacylglyceride level (not shown). Altogether, the above data suggested that CPTP could improve stress resistance ability including oxidative and heat stress in flies, which may lead to a prolonged lifespan.

### Chinese pond turtle peptide enhanced the endogenous antioxidant capacity in flies

**FIGURE 4:**
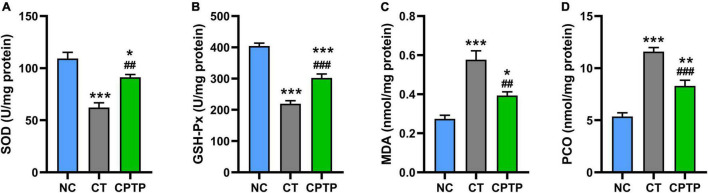
Chinese pond turtle peptide (CPTP) enhanced the antioxidant capacity in female flies. **(A)** Superoxide dismutase (SOD) activity; **(B)** GSH-Px activity; **(C)** Malondialdehyde (MDA) content; and **(D)** Protein carbonyl (PCO) content. *n* = 180–210 flies per group. The data are presented as mean ± SEM. Statistical test: two-tailed unpaired *t*-test (*P < 0.05, **P < 0.01, and ***P < 0.001 vs. NC group; ^##^*P* < 0.01 and ^###^*P* < 0.001 vs. CT group).

Natural aging would cause antioxidant system dysfunction, toxic peroxidation product accumulation, and consequently accelerate the death of organisms (46, 47). In aging organisms, it is commonly observed that there is a remarkable decline in the activities of peroxidase and SOD whereas an increase in the levels of lipid peroxidation and protein carbonylation (48). SOD and GSH-Px are important antioxidant enzymes to inhibit the formation of free radicals and commonly serve as the first line of defense against reactive oxygen species during oxidative stress status (49). MDA is the most important product of membrane lipid peroxidation, which causes cytotoxic cross-linking polymerization of macromolecules such as proteins and nucleic acids, while PCO is the carbonyl product of oxidative damage of proteins (50). The activities of antioxidant enzymes in the CT group were significantly decreased than those in the NC group, reflecting the decreased function of the antioxidant system during natural aging in this study (Figures 4A,B). Comparatively, the levels of MDA and PCO of flies in the CT group were remarkably higher than those in the NC group (Figures 4C,D). However, when flies were given CPTP, the SOD and GSH-Px activities were increased by 46.85% (*P* < 0.01) and 37.56% (*P* < 0.001), while the MDA and PCO contents reduced by 31.70% (*P* < 0.01) and 28.29% (*P* < 0.001), as compared with the CT group (Figures 4A–D). Similarly, growing studies confirmed that bioactive peptides could increase the SOD and GSH-Px activities, as well as decrease the MDA and PCO levels (13, 16, 39). Therefore, CPTP prolonged the lifespan possibly as the result of the increasing activities of antioxidant-related enzymes and the reducing accumulation of peroxide products in aged flies.

### Chinese pond turtle peptide regulated the Nrf2/Keap1 signaling pathway

**FIGURE 5:**
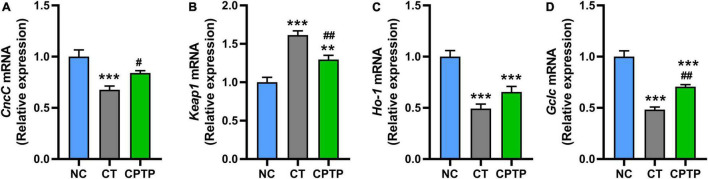
Chinese pond turtle peptide (CPTP) upregulated the Nfr2-mediated signaling pathway in female flies. The gene expression level of **(A)** *CncC*; **(B)** *Keap1*; **(C)** *Ho-1*; and **(D)** *Gclc*. *n* = 180–210 flies per group. The data are presented as mean ± SEM. Statistical test: two-tailed unpaired *t*-test (*P < 0.05, **P < 0.01, and ***P < 0.001 vs. NC group; ^#^*P* < 0.05 and ^##^*P* < 0.01 vs. CT group).

The abovementioned results show that CPTP increased the activity of antioxidant enzymes and reduced the accumulation of peroxide products in aged flies, which means that the prolonged longevity of CPTP might be due to oxidative stress attenuation. Nrf2/Keap1 signaling pathway could regulate the tolerance to oxidative stress and lifespan in flies (51). Furthermore, the upregulation of Nrf2 gene expression has the potential to remarkably improve the aging-related diseases and/or delay the aging process (5). Therefore, we then investigated whether CPTP could stimulate the Nrf2-mediated signaling pathway. First, the mRNA levels of *CncC* (the fly homologues of mammalian Nrf2) and its classical inhibitor Keap1 in flies were measured. The mRNA relative expressions of *Keap1* and *CncC* in flies in the CT group were significantly, respectively, increased and decreased compared with the NC group (Figures 5A,B). Compared with the CT group, CPTP supplementation significantly downregulated and upregulated the *Keap1* (*P* < 0.01) and *CncC* (*P* < 0.05) gene expression levels (Figures 5A,B), respectively. The mRNA levels of *Nrf2*’s downstream target genes, *Ho-1* and *Gclc*, were then used to determine its transcriptional activity. Ho-1 is a rate-limiting enzyme catalyzing the process of heme degradation to carbon monoxide and biliverdin, which are the precursors of bilirubin (a strong antioxidant) (52). Encouragingly, the mRNA levels of *Ho-1* and *Gclc* were markedly upregulated (*P* = 0.1142 and *P* < 0.001) after CPTP treatment (Figures 5C,D). Similarly, food-derived peptides from other sources such as soft-shelled turtle, cottonseed proteins, and okra can also ameliorate oxidative damage, aging, diabetes, and other aging-related diseases through the Nrf2 signaling pathway (19, 20, 53). Collectively, these findings suggested that CPTP could activate the Nrf2-mediated signaling pathway during the aging process, thereby enhancing the antioxidant capacity of flies and leading to an extended lifespan.

### Binding capacity to Keap1 of Chinese pond turtle peptide

The bioactivities of peptides are closely related to their molecular weight distribution, peptide chain length, amino acid composition, and peptide sequence. In the results of aforementioned above, CPTP has strong antioxidant and antiaging activities, which may be due to its high content of antioxidant peptides. Therefore, the following aspects of CPTP were analyzed: molecular weight distribution, amino acid composition, and peptide sequences. As shown in Supplementary Table 1, CPTP was mainly composed of small peptides below 1,000 Da, accounting for 94.71% of peptides. These small peptides could interact with free radicals more efficiently through their properties of easier to touch the intestinal barrier *in vivo* (49). Also, similar results have also been found in crimson snapper peptides (39), royal jelly-collagen peptide (46), sea cucumber protein hydrolysate (54), and rice protein hydrolysates (55), indicating that a lower molecular weight peptide exhibits higher antioxidant and antiaging activities. More importantly, some amino acid residues are the active sites of antioxidant peptides. The amino acids composition of CPTP is shown in Supplementary Table 2. Notably, sixteen amino acids were found in CPTP with a total content of 74.49 g per 100 g, and glycine and glutamic were the major amino acids, which is in line with our previous findings (22). Regarding the amino acid composition of peptides, Tyr, Try, Cys, Met, and His may act as hydrogen donors, whereas Asp and Glu can chelate metal ions. Furthermore, Ala, Val, Pro, Phe, and Leu may help to improve the solubility of peptides in the lipid phase and then facilitate interactions between the peptide and lipid-free radicals, thereby enhancing the antioxidant activity of peptides (56). In our study, the total content of the aforementioned major antioxidative amino acids was 44.95 g per 100 g, which made up 60.34% of CPTP. Then, the peptide sequence was identified by *de novo* sequencing. In total, 121 peptide sequences were identified in CPTP, and they were labeled as #1–121 based on the ALC (Supplementary Table 3). The molecular masses of these sequences ranged from 385.1961 to 823.3898 Da for CPTP, which were made up of 4–7 amino acid residues.

**FIGURE 6:**
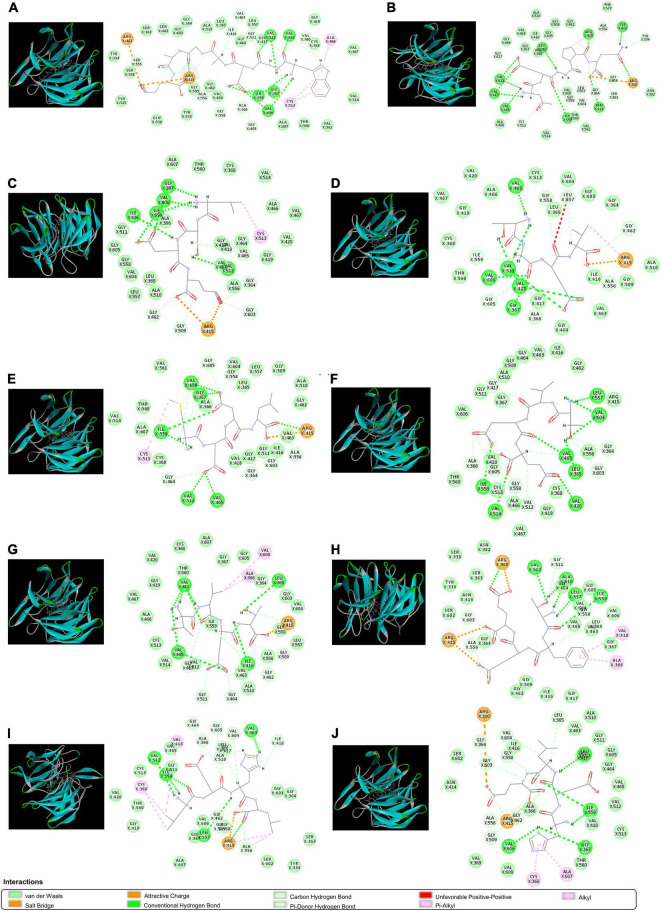
The 3D and 2D molecular interactions of peptides with the active site of Keap1. **(A)** #25; **(B)** #116; **(C)** #53; **(D)** #84; **(E)** #33; **(F)** #18; **(G)** #90; **(H)** #30; **(I)** #96; and **(J)** #62.

The ETGE sequence comprising a fragment of Nrf2 has been shown to be the essential motif in forming the binding with the Kelch domain of Keap1. The residues of Arg380, Arg415, Arg483, Ser363, Ser508, Ser555, Ser602, Asn382, Asn387, Tyr334, Tyr525, Tyr572, Phe577, Gln530, and His 436 are critical in Keap1-Nrf2 interaction (57). Therefore, molecular docking was used to screen for peptides from CPTP showing antioxidant activity by assessing the binding capacity of peptides to Keap1. As shown in Supplementary Table 3, 108 peptides in CPTP were successfully docking with a proportion of 89.23% among all identified sequences. Table 2 and Figure 6 show the top ten peptides with the highest (-) CDOCKER energy value. Moreover, there were 4 weak interactions observed from the docking with Keap1, i.e., hydrogen bonds (including conventional hydrogen bond, carbon-hydrogen, and Pi-donor hydrogen bond), van der Waals, hydrophobic (Pi-alkyl and alkyl), and electrostatic interactions (salt bridge and attractive charge) between the peptides, and the active site of Keap1 (Figure 6). #25 peptide that was composed of 16 residues had 24 hydrogen bonds with Ser555, Arg415, Ile416, Val463, Gly511, Val512, Val418, Val465, Cys368, Gly558, Ile559, Ala366, Gly605, Val606, Gly367, and Ala556. There were 24 van der Walls with Tyr525, Ser508, Tyr334, Ser363, Ser602, Gly603, Gly364, Ala510, Leu365, Gly464, Leu557, Gly417, Gly419, Cys368, Val467, Val514, Val561, Thr560, Ala607, Val604, Gly462, Gly509, Thr572, and Gln530. As for the hydrophobic and electrostatic interactions, #25 had 2 hydrophobic interactions with Arg483 and Arg415, while 3 electrostatic interactions with Ala466 and Cys513. Among them, the residues of Ser555, Arg415, Tyr525, Ser508, Tyr334, Ser363, Ser602, Gln530, and Arg483 are explicit key residues of Nrf2-Keap1 interaction. Similarly, #116 had 24 hydrogen bonds, 16 van der Walls, 1 hydrophobic interaction, and 1 electrostatic interaction with the active site of Keap1 (including Arg415, Ser602, Tyr334, Arg380, Ser363, Phe577, and Asn382). #87 had 15 hydrogen bonds, 20 van der Walls, 1 hydrophobic interaction, and 2 electrostatic interactions with the active site of Keap1 (including Arg415). #84 had 15 hydrogen bonds, 17 van der Walls, 1 hydrophobic interaction, and 1 electrostatic interaction with the active site of Keap1 (including Arg415). #33 had 15 hydrogen bonds, 17 van der Walls, 1 hydrophobic interaction, and 1 electrostatic interaction with the active site of Keap1 (including Arg415). #18 had 17 hydrogen bonds and 18 van der Walls with the active site of Keap1 (including Arg415). #90 had 16 hydrogen bonds, 21 van der Walls, 2 hydrophobic interactions, and 3 electrostatic interactions with the active site of Keap1 (including Arg415). #30 had 13 hydrogen bonds, 18 van der Walls, 2 hydrophobic interactions, and 3 electrostatic interactions with the active site of Keap1 (including Arg415, Arg380, Ser602, Tyr334, Ser363, and Asn382). #96 had 19 hydrogen bonds, 16 van der Walls, 5 hydrophobic interactions, and 1 electrostatic interaction with the active site of Keap1 (including Arg415, Ser363, Tyr334, and Ser602). #62 had 16 hydrogen bonds, 17 van der Walls, 2 hydrophobic interactions, and 2 electrostatic interactions with the active site of Keap1 (including Arg415, Ser602, and Arg380). In a nutshell, the contacts of the aforementioned representative peptide sequences identified from CPTP to the residues (Arg380, Arg415, Arg483, Ser363, Ser508, Ser555, Ser602, Tyr334, Tyr525, Asn382, Gln530, Phe577) suggested that CPTP has the potential to competitively bind to Keap1, stimulate the biological activities of Nrf2, and further displayed its antioxidant activity by regulating the Nrf2/Keap1 signaling pathway.

**TABLE 2:** Information of the top 10 identified peptides from *de novo* sequencing with the highest (-) CDOCKER energy in Chinese pond turtle peptide (CPTP).

No.	Sequence	ALC (%)	Mass (Da)	(-) CDOCKER energy (kcal/mol)
#25	WGDAGAE	94.8	704.2766	139.848
#116	NEGPQ	90.2	543.2289	111.606
#53	LSEE	93.5	476.2118	106.728
#84	TGEV	91.7	404.1907	106.029
#33	M(+42.01)DDL	94.4	534.1996	104.147
#18	TVEE	95.5	476.2118	102.252
#90	TVET	91.3	448.2169	101.389
#30	TFEE	94.5	524.2119	99.6856
#96	LEHL	91	510.2802	99.04
#62	HELE	92.7	526.2387	98.9327

## Conclusion

**FIGURE 7:**
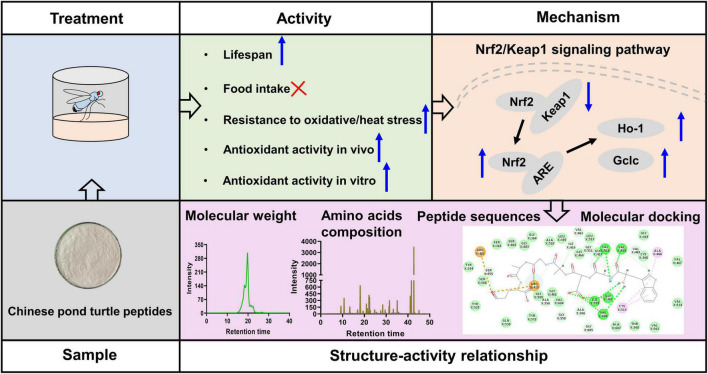
Antioxidant and antiaging effects of Chinese pond turtle peptide (CPTP) *via* the Nrf2/Keap1 signaling pathway in mice.

In this study, CPTP exhibited strong free radical scavenging activities revealed by DPPH, ABTS, and hydroxy. Subsequently, the experiment on the antiaging effect of CPTP revealed that CPTP significantly extended the lifespan of female flies but not reduced their food intake. Simultaneously, CPTP significantly improved stress resistances of oxidative and heat stress but exerted no protective effect against starvation stress. Mechanistically, CPTP activated the Nrf2/Keap1 signaling pathway, thereby slowing down the decline in antioxidant defense capacity of flies as time went by. Intriguingly, the representative ten peptide sequences could actively interact with the binding sites of Keap1 such as Arg380, Arg415, Arg483, Ser363, Ser508, Ser555, Ser602, Tyr334, Tyr525, Asn382, Gln530, and Phe577. Collectively, our results indicated that CPTPs could be a good source for antioxidant and antiaging agents (Figure 7), and our further determination of bioactive peptides with Keap1 inhibitory capability could pave the way for the future peptide purification of CPTP.

## Data availability statement

The original contributions presented in this study are included in the article/Supplementary Material, further inquiries can be directed to the corresponding authors.

## Author contributions

QW, HZ, and FF designed the study and reviewed the manuscript. QW, ZY, JCZ, JHZ, and FS performed the experiment. PY provided the material. QW and ZY analyzed the data. QW wrote the manuscript. All authors contributed to the article and approved the submitted version.

## Conflict of interest

The authors declare that the research was conducted in the absence of any commercial or financial relationships that could be construed as a potential conflict of interest.

## Publisher’s note

All claims expressed in this article are solely those of the authors and do not necessarily represent those of their affiliated organizations, or those of the publisher, the editors and the reviewers. Any product that may be evaluated in this article, or claim that may be made by its manufacturer, is not guaranteed or endorsed by the publisher.
