# Supplementary material for: Antiaging function of Chinese pond turtle (Chinemys reevesii) peptide through activation of the Nrf2/Keap1 signaling pathway and its structure-activity relationship
Source: Front Nutr. 2022 Jul 22;9:961922. doi: 10.3389/fnut.2022.961922 (PMC9355154; doi:10.3389/fnut.2022.961922)
Supplement: Supplementary file 1 [file Data_Sheet_1.docx]

Supplementary Material

**Supplementary Table 1.** The molecular weight distribution of CPTP.

| Molecular weight range (Da) | > 1000 | 500-1000 | 180-500 | < 180 |
| --- | --- | --- | --- | --- |
| Content (%) | 5.29 ± 0.08 | 22.99 ± 0.77 | 60.39 ± 1.01 | 11.32 ± 0.20 |

**Supplementary Table 2.** The amino acid composition of CPTP.

| Amino acid | Content (g/100g peptide) | Amino acid | Content (g/100g peptide) |
| --- | --- | --- | --- |
| Asp | 5.99 ± 0.42 | Pro | 7.24 ± 0.94 |
| Glu | 10.95 ± 0.89 | Tyr | 2.06 ± 0.62 |
| Ser | 2.09 ± 0.11 | Val | 3.59 ± 0.59 |
| Gly | 11.76 ± 1.24 | Met | 1.42 ± 0.22 |
| His | 0.92 ± 0.12 | Ile | 5.29 ± 0.57 |
| Arg | 3.13 ± 0.52 | Leu | 4.89 ± 0.48 |
| Thr | 2.21 ± 0.29 | Phe | 2.29 ± 0.31 |
| Ala | 5.60 ± 0.83 | Lys | 5.06 ± 0.65 |

**Supplementary Table 3.** Information of the peptides identified from *de novo* sequencing in CPTP and their (-) CDOCKER energy to Keap1.

| No. | Sequence | ALC (%) | Mass (Da) | (-) CDOCKER Energy (kcal/mol) | No. | Sequence | ALC (%) | Mass (Da) | (-) CDOCKER Energy (kcal/mol) |
| --- | --- | --- | --- | --- | --- | --- | --- | --- | --- |
| #1 | TKLPK | 98.6 | 585.3849 | 42.8006 | #62 | HELE | 92.7 | 526.2387 | 98.9327 |
| #2 | ALRLP | 98.5 | 568.3696 | F | #63 | TN(+0.98)APPH | 92.6 | 636.2867 | 67.9148 |
| #3 | PPLPK | 98.2 | 550.3478 | 29.3168 | #64 | LN(+0.98)HL | 92.6 | 496.2645 | 87.255 |
| #4 | A(+42.01)GPR | 98.1 | 441.2336 | 68.522 | #65 | TPGAM | 92.5 | 475.2101 | 78.5768 |
| #5 | YGLPR | 97.2 | 604.3333 | 60.5982 | #66 | TEPPK | 92.4 | 570.3013 | 68.7593 |
| #6 | M(+42.01)DAL | 97.1 | 490.2097 | 89.227 | #67 | M(+42.01)LPN | 92.4 | 515.2414 | 60.1936 |
| #7 | LGPR | 97 | 441.27 | 74.1931 | #68 | M(+42.01)DALVK | 92.4 | 717.3731 | 45.9504 |
| #8 | TPEM | 96.9 | 476.1941 | 79.3738 | #69 | GLHET | 92.4 | 555.2653 | 63.254 |
| #9 | TPKPE | 96.8 | 570.3013 | -133.468 | #70 | LLVDV | 92.3 | 557.3425 | 68.6431 |
| #10 | TPEE | 96.8 | 474.1962 | 77.8204 | #71 | TPSPL | 92.2 | 513.2798 | 60.3361 |
| #11 | LKYP | 96.7 | 519.3057 | 47.4806 | #72 | M(+42.01)(+15.99)LPV | 92.2 | 516.2618 | 58.5432 |
| #12 | LDFPR | 96.6 | 646.3438 | 46.0852 | #73 | LN(+0.98)KL | 92.2 | 487.3006 | 90.7903 |
| #13 | WQPPR | 96.3 | 682.3551 | 42.7738 | #74 | TPLLA | 92 | 513.3162 | 3.11056 |
| #14 | VSLPR | 96.2 | 570.3489 | 34.0309 | #75 | TLEH | 92 | 498.2438 | 92.175 |
| #15 | TLGDE | 96.2 | 533.2333 | 56.0819 | #76 | LPVDF | 92 | 589.3112 | 68.0919 |
| #16 | LELPR | 95.9 | 626.3751 | 83.1389 | #77 | LN(+0.98)VPKP | 91.9 | 667.3904 | 51.0563 |
| #17 | TEMPPLH | 95.7 | 823.3898 | 64.8893 | #78 | KLEL | 91.9 | 501.3162 | 82.3053 |
| #18 | TVEE | 95.5 | 476.2118 | 102.252 | #79 | WLPV | 91.8 | 513.2951 | 43.1349 |
| #19 | RDLK | 95.4 | 530.3176 | 50.7352 | #80 | TGGEV | 91.8 | 461.2122 | F |
| #20 | LLDM | 95.3 | 490.2461 | 83.4007 | #81 | TDAL | 91.8 | 418.2064 | 89.971 |
| #21 | LFDR | 95.1 | 549.2911 | 80.1993 | #82 | SEAPPH | 91.8 | 636.2867 | F |
| #22 | MGPR | 95 | 459.2264 | 63.237 | #83 | LRDF | 91.8 | 549.2911 | 89.8012 |
| #23 | YKPDL | 94.9 | 634.3326 | 60.9057 | #84 | TGEV | 91.7 | 404.1907 | 106.029 |
| #24 | FEGFPK | 94.9 | 723.3591 | 70.2238 | #85 | LELP | 91.7 | 470.274 | 66.1 |
| #25 | WGDAGAE | 94.8 | 704.2766 | 139.848 | #86 | TTGL | 91.6 | 390.2114 | 91.1968 |
| #26 | TDKL | 94.8 | 475.2642 | 86.8506 | #87 | VGGPL | 91.5 | 441.2587 | 91.1968 |
| #27 | M(+42.01)DPL | 94.8 | 516.2254 | 66.0124 | #88 | V(+42.01)GPL | 91.5 | 426.2478 | F |
| #28 | SN(+0.98)LAH | 94.7 | 541.2496 | 83.3044 | #89 | LPKPL | 91.4 | 566.3792 | -20.3565 |
| #29 | WKPPV | 94.6 | 625.3588 | 35.4762 | #90 | TVET | 91.3 | 448.2169 | 101.389 |
| #30 | TFEE | 94.5 | 524.2119 | 99.6856 | #91 | FGHT | 91.2 | 460.207 | 98.0731 |
| #31 | FKDL | 94.5 | 521.2849 | 94.333 | #92 | EHLE | 91.2 | 526.2387 | 74.9161 |
| #32 | TEAP | 94.4 | 416.1907 | 92.2202 | #93 | VELPR | 91.1 | 612.3595 | 69.627 |
| #33 | M(+42.01)DDL | 94.4 | 534.1996 | 104.147 | #94 | LSLRP | 91.1 | 584.3646 | F |
| #34 | LPHL | 94.4 | 478.2903 | 66.5236 | #95 | LPGVDA | 91 | 570.3013 | 72.797 |
| #35 | AAGPA | 94.4 | 385.1961 | 74.1807 | #96 | LEHL | 91 | 510.2802 | 99.04 |
| #36 | VLLPK | 94.3 | 568.3948 | 20.4415 | #97 | TN(+0.98)VPPLH | 90.9 | 777.4021 | 45.5867 |
| #37 | VGFPK | 94.2 | 546.3166 | 63.3763 | #98 | TDVPPLH | 90.9 | 777.4021 | F |
| #38 | LLDHL | 94.2 | 609.3486 | 52.4713 | #99 | SEAPPHL | 90.9 | 749.3708 | F |
| #39 | KLEF | 94.2 | 535.3006 | 64.7242 | #100 | QEAPPH | 90.9 | 677.3133 | 76.2591 |
| #40 | TVVAPL | 94.1 | 598.369 | 69.8662 | #101 | LDRPF | 90.9 | 646.3438 | 44.9404 |
| #41 | TGVPK | 94.1 | 500.2958 | 75.3594 | #102 | PVVP | 90.8 | 410.2529 | F |
| #42 | KLPF | 94 | 503.3108 | 61.3817 | #103 | TVDT | 90.7 | 434.2013 | 97.5143 |
| #43 | YDLDF | 93.8 | 671.2802 | 94.7477 | #104 | TDVPPHL | 90.7 | 777.4021 | 60.6931 |
| #44 | PAWE | 93.8 | 501.2223 | 58.7565 | #105 | LEGDL | 90.7 | 545.2697 | 95.1745 |
| #45 | FKDF | 93.8 | 555.2693 | -49.2549 | #106 | APVLDV | 90.7 | 612.3483 | 27.7853 |
| #46 | ELLPAE | 93.8 | 670.3537 | 50.8922 | #107 | AAPF | 90.7 | 404.206 | F |
| #47 | TLDE | 93.7 | 476.2118 | 87.1499 | #108 | A(+42.01)APL | 90.7 | 412.2322 | 60.7177 |
| #48 | YFPH | 93.6 | 562.254 | -26.2974 | #109 | YKEFG | 90.6 | 642.3013 | F |
| #49 | VEAPLPK | 93.6 | 752.4432 | 85.5393 | #110 | TN(+0.98)VPPHL | 90.6 | 777.4021 | 39.1004 |
| #50 | TLHE | 93.6 | 498.2438 | 88.985 | #111 | NFDF | 90.6 | 541.2172 | 97.3275 |
| #51 | LEVPH | 93.6 | 593.3173 | 68.8028 | #112 | K(+42.01)FPS | 90.6 | 519.2693 | 62.3429 |
| #52 | FGEK | 93.6 | 479.238 | F | #113 | EGGFDF | 90.4 | 670.2598 | 90.1761 |
| #53 | LSEE | 93.5 | 476.2118 | 106.728 | #114 | TDAPPH | 90.3 | 636.2867 | 71.188 |
| #54 | VAGPR | 93.3 | 498.2914 | 75.0997 | #115 | VGPW | 90.2 | 457.2325 | 71.7541 |
| #55 | FRDL | 93.3 | 549.2911 | 78.1117 | #116 | NEGPQ | 90.2 | 543.2289 | 111.606 |
| #56 | TYDSL | 93.1 | 597.2646 | 80.9008 | #117 | LQLE | 90.2 | 501.2798 | -17.8426 |
| #57 | LLDA | 93.1 | 430.2427 | 90.215 | #118 | K(+42.01)WEN | 90.2 | 617.2809 | -111.457 |
| #58 | TGVE | 92.9 | 404.1907 | 92.3051 | #119 | ALPF | 90.1 | 446.2529 | F |
| #59 | TLDEL | 92.8 | 589.2959 | -5.75863 | #120 | AFPLP | 90.1 | 543.3057 | -33.6609 |
| #60 | AVFPR | 92.8 | 588.3384 | -11.7097 | #121 | PPFT | 90 | 460.2322 | F |
| #61 | VDFPR | 92.7 | 632.3282 | 17.7021 |  |  |  |  |  |
